# Effect of Molecular Weight on Gelling and Viscoelastic Properties of Poly(caprolactone)–b-Poly(ethylene glycol)–b-Poly(caprolactone) (PCL–PEG–PCL) Hydrogels

**DOI:** 10.3390/polym12102372

**Published:** 2020-10-15

**Authors:** Noam Y. Steinman, Noam Y. Bentolila, Abraham J. Domb

**Affiliations:** The Alex Grass Center for Drug Design and Synthesis and Center for Cannabis Research and the Institute of Drug Research, School of Pharmacy-Faculty of Medicine, The Hebrew University of Jerusalem, Jerusalem 91120, Israel; Noam.Steinman@mail.huji.ac.il (N.Y.S.); noambent@gmail.com (N.Y.B.)

**Keywords:** injectable hydrogels, PEG–PCL, pseudoplastic

## Abstract

Hydrogels based on poly(caprolactone)–*b*-poly(ethylene glycol)–*b*-poly(caprolactone) (PCL–PEG–PCL) have been evaluated extensively as potential injectable fillers or depots for controlled release of drugs. Common drawbacks of these copolymer systems include instability of aqueous solutions and low mechanical strength of gels, issues which are commonly overcome by adding pendant groups to the end of the copolymer chains. Here, a systematic study of the effects of increasing polymer molecular weight (MW) is presented, utilizing PEG blocks of MW 2, 4 or 8 kDa. Triblock copolymers were prepared by the ring-opening polymerization of Ɛ-caprolactone by PEG. Copolymers prepared with PEG MW 2 kDa did not form hydrogels at any copolymer molecular weight. Copolymers prepared with PEG MW 4 kDa formed gels at MW between 11 and 13.5 kDa, and copolymers prepared with PEG MW 8 kDa formed gels at MW between 16 and 18 kDa. Copolymers with PEG block 8 kDa formed hydrogels with high viscosity (17,000 Pa·s) and mechanical strength (G′ = 14,000 Pa). The increased gel strength afforded by increased molecular weight represents a simple modification of the reactants used in the reaction feed without added synthetic or purification steps. Shear-thinning of PCL-PEG-PCL triblock copolymer hydrogels allowed for injection through a standard 23G syringe, allowing for potential use as dermal fillers or drug delivery depots.

## 1. Introduction

Biocompatible and biodegradable polymers are ubiquitous across modern medicine [1], finding their most common use as implants [2,3] or as drug delivery systems [4,5]. Decades of research have produced polymers that may be fine-tuned to match nearly any desired application [6]. Some polymers may form hydrogels, three-dimensional systems which are able to retain large quantities of water due to either chemical or physical crosslinking, rendering these polymers attractive platforms for dermal fillers or for the delivery of hydrophilic drugs [7,8]. The ability to inject a polymeric material through a syringe and form a hydrogel post-injection is advantageous, as formation of a hydrogel depot may afford localized release of therapeutic agents or provide tissue augmentation [9,10].

Triblock copolymers of poly(ethylene glycol) (PEG) and poly(caprolactone) (PCL) have been studied extensively as injectable depot-forming hydrogels [11]. The hydrophilic PEG block and hydrophobic PCL blocks in the PCL–PEG–PCL triblock copolymer combine to form temperature-dependent physical crosslinking which affords a thermoresponsive hydrogel [12]. The advantages of using PCL as the hydrophobic block instead of other biocompatible polyesters such as poly(lactic-co-glycolic acid) (PLGA) include long shelf-life and enhanced viscoelastic properties and mechanical strength [13]. In addition, the powdery morphology of the copolymer with PEG is far easier to handle than the pasty copolymers of PEG with PLGA [14]. Nevertheless, several drawbacks to the applicability of PCL–PEG–PCL triblock copolymers remain, and a study of the effect of high molecular weight has, to the best of our knowledge, yet to be reported. High molecular weight polymers may reduce the gelation temperature required to form the hydrogel, thus allowing the possibility of injection as a liquid and subsequent formation of a hydrogel post-injection, as is commonly employed with the triblock copolymer analogue with PLGA [15]. Furthermore, the crystallinity of PCL does not allow for a stable aqueous solution, and the relatively short PCL blocks render a short shelf-life of the polymer [16,17]. We therefore report a systematic study of the effect of increased polymer molecular weight on the gelling properties of PCL–PEG–PCL triblock copolymer hydrogels, rather than the more commonly used pendant groups to the parent triblock structure in order to reduce both crystallinity and gelling temperature [18,19,20,21,22].

A simple adjustment of polymer block molecular weight afforded strong, viscous hydrogels which were easy to prepare and may be injected as a hydrogel using standard equipment due to shear-thinning experienced in the syringe upon injection. As a result of increased copolymer molecular weight (MW), the injectable hydrogels were stable at room temperature for over nine months and at physiological temperature for over one month.

## 2. Materials and Methods

### 2.1. Materials

PEG 2 kDa, PEG 4 kDa and stannous octoate were purchased from Simga Aldrich (Rehovot, Israel). Ɛ-caprolactone was purchased from Tzamal D-Chem (Petach Tikva, Israel). PEG 8 kDa was purchased from Holland Moran (Yehud, Israel). Solvents were of analytical grade from Biolab (Jerusalem, Israel).

### 2.2. General Methods

Chemical reactions were performed in disposable 4-mL glass vials equipped with magnetic stirrers and polypropylene caps with silicone septa under N_2_ gas. ^1^H NMR spectra were obtained on a Varian 300 MHz spectrometer with CDCl_3_ as the solvent and tetramethylsilane as the shift reference. The molecular weights of the polymers were estimated using a gel permeation chromatography system consisting of a Waters 1515 Isocratic HPLC pump with a Waters 2410 Refractive Index Detector and a Rheodyne (Cotati, CA, USA) injection valve with a 20 mL loop (Waters, Milford, MA, USA). Samples were eluted with CHCl_3_ through a linear Styragel HR4E column (7.8 × 300 mm i.d.; Waters) at a flow rate of 1 mL/min. The molecular weights were determined relative to polystyrene standards (Polyscience, Warrington, PA, USA) using a Breeze computer program. Fourier transform infrared spectroscopy analysis was performed using a Smart iTR sampling accessory for a Nicolet iS10 spectrometer with a diamond crystal.

### 2.3. Synthesis

PCL–PEG–PCL triblock copolymers were synthesized by ring-opening polymerization (ROP) of Ɛ-caprolactone (CL) by poly(ethylene glycol) (PEG) diol. In each reaction, PEG of MW 2, 4 or 8 kDa was used. A sample synthesis was as follows: 50 µL of a 0.33 g mL^−1^ solution of stannous octoate in dichloromethane was added to a melt of PEG 8 kDa (1.4 g, 0.18 mmo) and CL (2.1 g, 18 mmol) purged with N_2_. Solvent was allowed to evaporate, and the vial was again purged with N_2_. The mixture was stirred at 120 °C for 2 h, and then overnight at 150 °C. The crude product was washed with ethanol and dried to afford PCL–PEG–PCL triblock copolymer (Entry **X**) in quantitative yield. ^1^H NMR (300 MHz, CDCl_3_): δ 3.99 (t, *J* = 6 Hz, 2H), 3.58 (s, 2.6H, PEG), 2.24 (t, *J* = 9 Hz, 2H), 1.63–1.53 (m, 4H), 1.36–1.26 (m, 2H). IR: ν = 2944 (s), 2865 (s), 1722 (s), 1470 (w), 1419 (w), 1397 (w), 1366 (w), 1342 (w), 1294 (w), 1240 (s), 1188 (s), 1106 (s), 1062 (w), 1045 (w), 961 (s), 842 (s), 732 (s).

Polymers were prepared by choosing one PEG (2, 4 or 8 kDa) as diol initiator and mixing at various feed ratios with CL.

### 2.4. Gel Studies

Aqueous polymer solutions were prepared with 30% (*w*/*v*) polymer in double-distilled water by mixing at 4 °C until homogeneous. If overnight mixing at 4 °C did not afford a homogeneous mixture, the polymer was deemed insoluble at this concentration. Gelling temperatures were measured by incubating the solution at a given temperature for 10 min, followed by inversion of the vial to test for gelling. If the gel did not flow, the temperature was recorded as the gelling temperature of the solution (T_gel_). Results are accurate to +/−2.0 °C [15].

### 2.5. Rheological Studies

Polymer viscosity was measured by rotational tests on a Physica MCR 101 rheometer (Anton Paar, Austria) as a function of shear rate, and viscoelastic properties were measured by oscillation tests on the same instrument. Measurements were performed at shear rates from 0.01 to 100 s^−1^ at 25 °C.

## 3. Results

### 3.1. Synthesis

Gelling behavior and viscoelastic properties of poly(caprolactone)–*b*-poly(ethylene glycol)–*b*-poly(caprolactone) (PCL–PEG–PCL) hydrogels are influenced by the molecular weight (MW) of each polymer block [23]. To study the behavior of high molecular weight copolymers, the PEG macroinitiator was increased using various PEG block MWs (2, 4, or 8 kDa, Table 1). Polymers were prepared by ring-opening polymerization (ROP) of caprolactone (CL) by PEG diol with a stannous octoate catalyst. We chose PEG and PCL for the respective hydrophilic and hydrophobic polymer blocks in order to effect amphiphilicity, a key requirement for the preparation of physically crosslinked hydrogels [24]. Furthermore, PCL was chosen for the hydrophobic block due to its relatively slow hydrolytic degradation and for its high mechanical strength [25]. Unique polymers within each series were prepared by controlling the relative amount of CL monomer in the feed. Powdery polymers were obtained in quantitative yields, since homogeneous polymers were obtained without need for further purification. No PEG was observed in the ethanol wash, indicating complete consumption of starting materials.

### 3.2. Characterization

**Figure 1:**
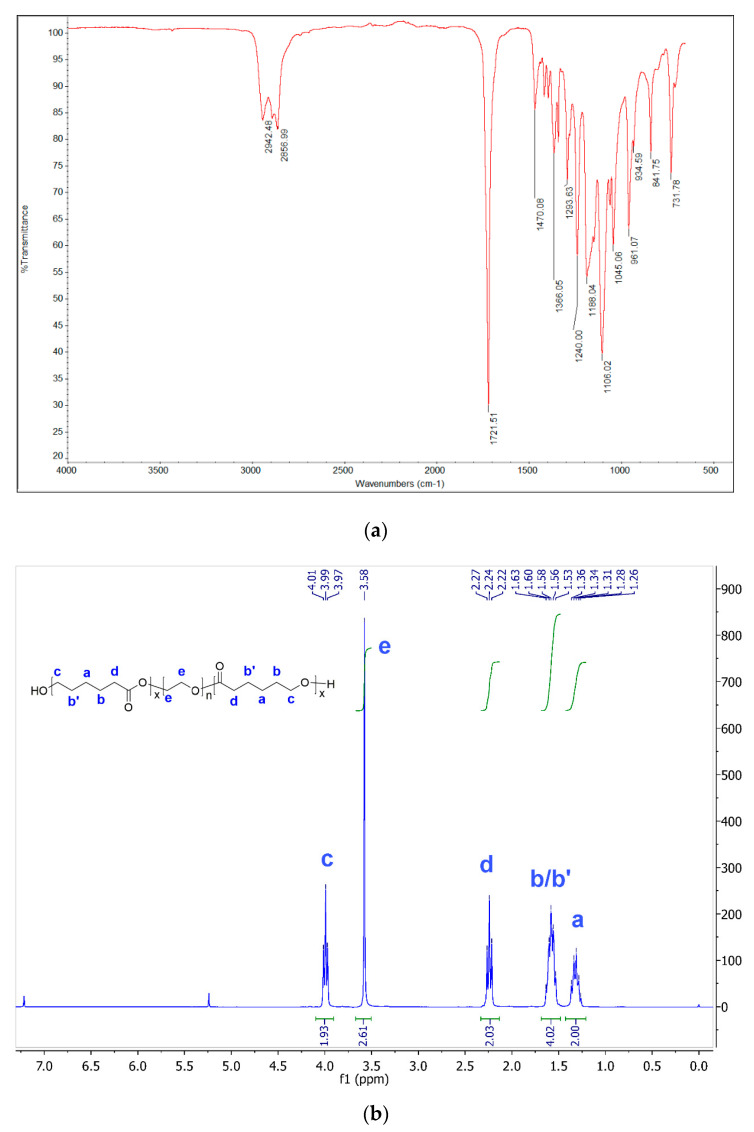
Structure of PCL–PEG–PCL triblock copolymers prepared by ring-opening polymerization of Ɛ-caprolactone by poly(ethylene glycol) diol with stannous octoate catalyst. (**a**) FT-IR spectrum of entry **X** showing characteristic ester stretch at 1721 cm^−1^; (**b**) ^1^H NMR spectrum of entry **X** with peak assignments.

The chemical structure of PCL–PEG–PCL triblock copolymers was confirmed spectroscopically by appearance of a characteristic ester C=O bend between 1731 and 1721 cm^−1^ in the infrared (IR) spectra (Figure 1a). COO and C–O–C stretch vibrations at 1240 and 1106 cm^−1^, respectively, further confirmed the chemical connectivity between polymer blocks [26]. The Proton Nuclear Magnetic Resonance (^1^H NMR) spectra displayed the presence of PEG block as a singlet at δ 3.58, and PCL peaks in their characteristic chemical shifts (Figure 1b).

### 3.3. Molecular Weight Determination

PCL–PEG–PCL triblock copolymers were prepared in various molecular weights by modifying the PEG macroinitiator molecular weight as well as the relative amount of CL monomer in the feed. The weight average (M_w_) and number average (M_n_) molecular weights were estimated by gel permeation chromatography (GPC), and polydispersity (PDI) was calculated from these values. Furthermore, unimodal peaks with significantly shorter retention times than PEG starting materials indicated consumption of the macroinitiator. M_n_ was calculated by comparing ^1^H NMR peak integrations of PEG and PCL polymer blocks, normalized by supplier MW data for PEG blocks. In most cases, M_n_ data as calculated by ^1^H NMR closely resembled the GPC estimate and reflected the expected chain length as calculated based on the feed of starting materials. The PDI values provided by GPC (< 2) indicated polymers with low polydispersity.

Full spectral data, including GPC chromatographs, are available as supplementary information (Figures S1–S28).

### 3.4. Gelling Properties

**Figure 2:**
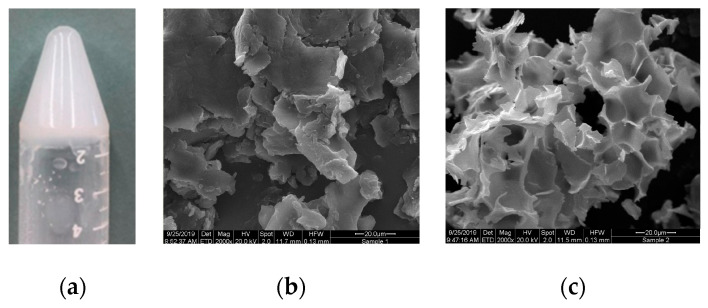
(**a**) PCL–PEG–PCL hydrogels were formed by increasing PEG block MW to 4 or 8 kDa; (**b**) scanning electron microscope (SEM) micrograph of a dehydrated polymer sample displays the 3D structure of the polymer without heating to form a gel; (**c**) the gel network resulting from heating at 50 °C for one minute.

For each polymer series (based on either PEG 2, 4 or 8 kDa), copolymer MW was varied by the amount of CL included in the feed, and its effect on the solution behavior of the resultant copolymer was studied. In order to isolate the effects of polymer structure on its gel properties, fixed 30% *w*/*v* aqueous solutions were prepared by mixing overnight at 4 °C until homogenous mixtures were observed. In general, low MW polymers prepared with the same MW PEG macroinitiator displayed solubility at all temperatures (4–80 °C), owing to the dominance of the hydrophilic PEG block. As MW was increased, the amphiphilic copolymer maintained solubility at cool temperatures, yet also formed a hard gel upon heating (Figure 2a). As MW was further increased, the hydrophobicity of the copolymer was increased, and so the polymer was no longer soluble at any temperature.

When low MW PEG (2 kDa) was used as the hydrophilic block, no gelling effect was observed, no matter the PCL block MW. As such, copolymers with MW below 4.9 kDa were freely soluble in aqueous media, and MW above 4.9 kDa afforded insoluble polymers. While the polymers had the required amphiphilicity for formation of hydrogels, they lacked sufficient polymer block MW for the formation of physical crosslinking upon heating.

In a previous report, we established that gelling properties of triblock thermoresponsive hydrogels are influenced by MW of the central PEG block [15]. In order to induce a gelling effect, we raised PEG MW to 4 kDa. Polymers with this increased hydrophilic PEG block MW formed opaque gels upon heating to 50 °C for one minute (Figure 2). Here, the trend of increasing MW was maintained: At low MW (<11 kDa), polymers remained in solution up to 80 °C. Once polymer MW was increased to between 11 and 13.5 kDa, the heated polymer solution formed a gel, which upon cooling to room temperature remained stable for over nine months. The polymer gels were stable at representative physiological temperatures for 14 weeks. Copolymer MW above 13.5 kDa induced overwhelming hydrophobicity of the PCL block, and the polymers were insoluble in aqueous media at any temperature (4–80 °C).

A similar trend was observed for PCL–PEG–PCL triblock copolymers prepared with a PEG block of 8 kDa. Copolymers with MW < 16 kDa were freely soluble in aqueous media due to the overwhelming hydrophilicity of PEG block. As MW was increased, copolymers with MW 16–18 kDa were soluble at cool temperatures, forming an opaque gel when heated to 25 °C. The lower T_gel_ may be attributed to the increase in hydrophobic PCL block MW, as lower temperatures were required to form the physical crosslinks which lead to gel formation. Once polymer MW was increased over 18 kDa, the polymer was insoluble at any temperature (4–80 °C).

### 3.5. Rheology Studies

**Figure 3:**
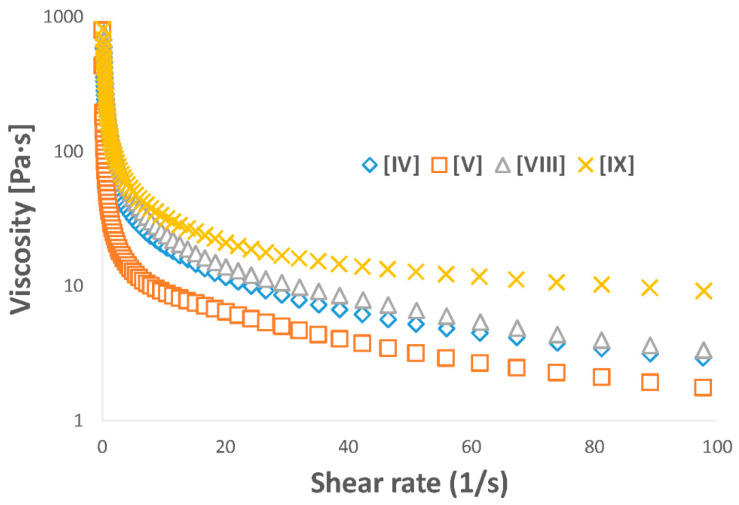
Pseudoplastic behavior of PCL–PEG–PCL hydrogels was displayed by the dramatic reduction in viscosity upon increased shear rate. High zero-shear viscosities are expected to provide immobility post-injection.

**Figure 4:**
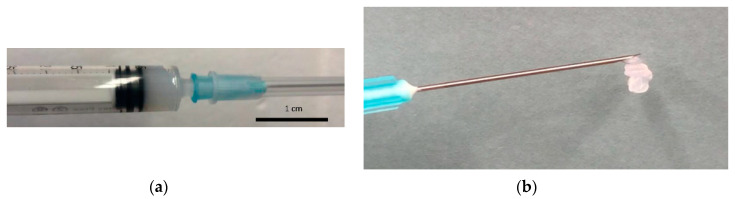
(**a**) PCL–PEG–PCL hydrogel was easily loaded into a syringe; (**b**) the gel was easily injected through a 23G syringe.

The flow behavior of hydrogels represents an important parameter in their application as dermal fillers or drug delivery systems. Accordingly, the viscosities of hydrogels based on polymers with PEG block MW 4 kDa (**IV** and **V**) and PEG block MW 8 kDa (**VIII** and **IX**) were measured with increasing shear. Zero-shear viscosity was measured up to 1300 Pa·s for **IV** and **V** and up to 17,000 for **VIII** and **IX**, indicating strong, viscous gels at rest state (Figure 3). The increase in viscosity as a result of overall polymer MW was characteristic of polymer solution behavior. The reduction of viscosity to <10 Pa·s upon application of shear displayed the pseudoplastic behavior necessary for injectability, as the pressure applied in a syringe was sufficient to allow the viscous gels to be injected through a 23G syringe (Figure 4). The increase in viscosity for higher MW polymers represents a distinct advantage for their potential application as immobile fillers or drug-delivery depots [27].

**Figure 5:**
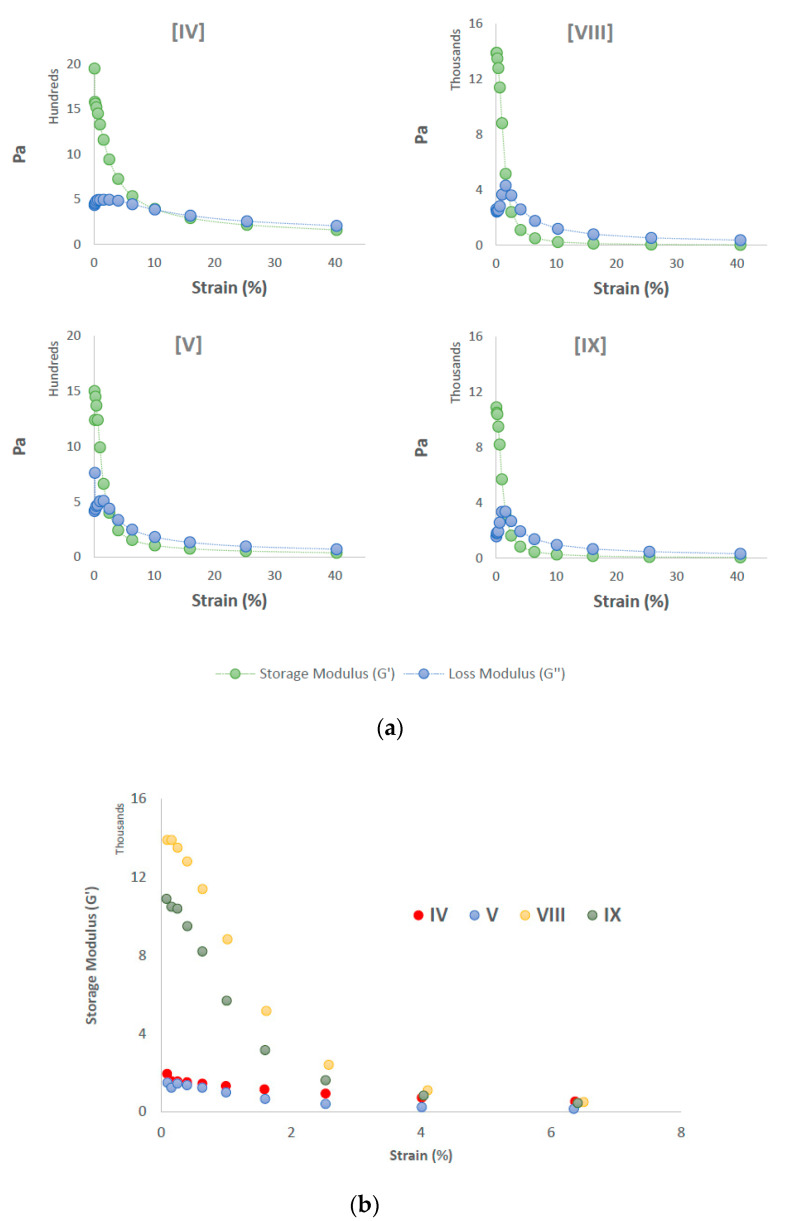
(**a**) Viscoelastic behavior of PCL–PEG–PCL hydrogels. All samples were viscoelastic solids at zero strain (G′ > G″); (**b**) storage moduli of hydrogels at low strain displayed increased strength of hydrogels based on PEG 8 kDa (**VIII, IX**) compared to those based on PEG 4 kDa (**IV, V**).

Oscillatory rheology of the copolymer hydrogels was measured to indicate the mechanical strength of the viscoelastic gels. In general, hydrogels based on PEG 8 kDa were stronger than those based on PEG 4 kDa, with zero-strain G′ approaching 14,000 Pa in **VIII** compared to just 2000 in **IV**. Indeed, at zero strain, the longer chain copolymer gels displayed more elastic behavior than lower MW copolymer gels (Figure 5). Upon application of shear, all hydrogels deformed into liquids, further evidence of pseudoplastic behavior, an important parameter in their application as injectable materials.

## 4. Discussion

Biodegradable polymeric hydrogels are commonly formed by temperature-dependent physical crosslinking of hydrophobic and hydrophilic polymer blocks. Strength, gelling temperature and stability of hydrogels may be tuned by both the identity and molecular weight (MW) of each polymer block [23]. Triblock copolymers of poly(d,l-lactic acid-co-glycolic acid)–*b*-poly(ethylene glycol)–*b*-poly(d,l-lactic acid-co-glycolic acid) (PLGA–PEG–PLGA) are a common example of such hydrogels, as the biodegradable polymer is able to form a temperature-dependent, physically crosslinked hydrogel network in aqueous media [15]. These hydrogels, however, have not displayed long-term stability, as gels begin to decompose in a matter of days [28]. In this work, we have replaced the hydrophobic PLGA blocks with poly(caprolactone) (PCL), a safe, biodegradable and hydrophobic polymer with relatively long degradation rates [29]. High molecular weight and low polydispersity of the triblock copolymers afforded the ability to form strong gels.

Several groups have reported PCL–PEG–PCL triblock copolymers based on low molecular weight PEG (<2 kDa) with sol–gel transition properties [12,30,31]. As biodegradable polymers with increased molecular weights are known to possess advantageous properties such as increased mechanical strength and longer degradation times [5,32], polymers were prepared with increasing PEG block MWs of 2, 4 or 8 kDa. By controlling polymer concentration, the range of PCL block MW required to induce gelling by hydrophobic interactions was defined for each PEG block MW.

Although soluble at ambient temperature, unmodified low MW PCL–PEG–PCL triblock copolymers form opaque gels when left to rest. This issue has commonly been overcome by extending the polymer chain with pendant groups such as cyclic ethers, polyesters or polycarbonates to prevent crystallization of PCL blocks [11,33]. The high MW polymers described here represent an alternative method to overcome PCL aggregation in solution. The increase in PEG block MW, and the appending of PCL blocks long enough to afford amphiphilicity required for hydrogel formation, afforded copolymers with high MW (>16 kDa) which were stable as gels for several months and did not aggregate unless heated.

A further consideration important to the application of injectable hydrogels as dermal fillers or drug delivery depots is their immobility post-injection. For this to be assured, hydrogels must possess high mechanical strength. In this study, injectable hydrogels of high molecular weight were prepared from PEG 8 kDa, affording triblock copolymers with MW > 16 kDa capable of forming hydrogels when dispersed in aqueous media. Whereas hydrogels prepared from polymers with lower MW (<14 kDa) displayed zero-shear G′ values of ~2000 Pa, relatively monodisperse polymers (PDI < 2) of high MW afforded hydrogels with zero-shear G′ values up to ~14,000 Pa, a 7-fold increase in solid state nature at rest. This enhanced mechanical strength is expected to promote immobility post-injection.

As an alternative to stimuli-responsive hydrogels, pseudoplastic behavior allows for temporary liquid state during injection due to the pressure applied to the gel inside the syringe, which upon injection returns to elastic or gel state [27]. All hydrogels described in this study displayed the shear-thinning required for applicability as injectable materials. Significantly, even high molecular weight polymers with increased mechanical strength exhibited pseudoplastic behavior and so represent potential candidates for dermal fillers or drug delivery depots due to their expected immobility post-injection. The shear-thinning experienced upon application of pressure in the syringe affords these gels with the capability of being easily injected to a desired site.

## 5. Conclusions

Triblock copolymers of PCL–PEG–PCL with molecular weights above 16 kDa afforded injectable hydrogels with high viscosities and mechanical strength. MW ranges for gel formation were determined for polymers based on PEG 4 or 8 kDa. Zero-shear viscosity of 17,000 Pa·s and G′ of 14,000 Pa were recorded for hydrogels based on PEG 8 kDa, indicating strong gels capable of maintaining immobility post-injection. Hydrogels were successfully loaded into and injected through a 23G syringe due to shear-thinning in the syringe during injection. This adjustment of molecular weight represents a simple modification resulting in strong, injectable pseudoplastic hydrogels capable of filling dermal cavities or releasing therapeutic materials at the injection site for several months.

## Figures and Tables

**Table 1:** Ten polymers, each with the general formula poly(caprolactone)–*b*-poly(ethylene glycol)–*b*-poly(caprolactone) (PCL–PEG–PCL), were prepared by ring-opening polymerization (ROP) of caprolactone (CL) by PEG diol.

Sample	PEG MW (kDa) ^1^	Expected Copolymer MW (kDa) ^2^	M_n_ (^1^H NMR, kDa) ^3^	M_n_ (GPC, kDa) ^4^	M_W_ (GPC, kDa) ^4^	PDI ^4^	Solubility ^5^	Gelling Temperature
I	2	3.0	3.0	4.1	4.8	1.2	Soluble	-
II	2	3.3	3.2	4.4	4.9	1.1	Insoluble	-
III	4	6.6	6.2	8.3	10.9	1.3	Soluble	-
IV	4	9.2	9.0	7.2	11.0	1.5	Soluble	50 °C
V	4	9.6	9.5	9.1	13.5	1.5	Soluble	50 °C
VI	4	10.0	9.7	10.2	15.0	1.5	Insoluble	-
VII	8	10.3	10.1	12.2	14.3	1.2	Soluble	-
VIII	8	16.8	16.8	9.1	16.3	1.8	Soluble	25 °C
IX	8	18.4	17.8	10.0	17.7	1.1	Soluble	25 °C
X	8	19.9	40.1	10.3	19.1	1.8	Insoluble	-

^1^ According to commercial source. ^2^ Calculated based on feed. ^3^ Determined by ^1^H NMR peak integrations. ^4^ Calculated from gel permeation chromatography (GPC). ^5^ Solubility in water at ambient temperature at 30% *w*/*v*.

## Supplementary Materials

The following are available online at https://www.mdpi.com/2073-4360/12/10/2372/s1, Figures S1–S9: ^1^H NMR spectra of polymers **I-IX**, Figures S10–S18: IR spectra of polymers **I-IX**.
