# Supplementary material for: Effect of Molecular Weight on Gelling and Viscoelastic Properties of Poly(caprolactone)–b-Poly(ethylene glycol)–b-Poly(caprolactone) (PCL–PEG–PCL) Hydrogels
Source: Polymers (Basel). 2020 Oct 15;12(10):2372. doi: 10.3390/polym12102372 (PMC7650642; doi:10.3390/polym12102372)
Supplement: Supplementary file 1 [file polymers-12-02372-s001.zip › polymers-969544-supplementary.docx]

**Supplementary Information**

Effect of Molecular Weight on Gelling and Viscoelastic Properties of Poly(caprolactone)–b-Poly(ethylene glycol)–b-Poly(caprolactone) (PCL–PEG–PCL) Hydrogels

Noam Y. Steinman, Noam Y. Bentolila and Abraham J. Domb *

The Alex Grass Center for Drug Design and Synthesis and Center for Cannabis Research and the Institute of Drug Research, School of Pharmacy-Faculty of Medicine, The Hebrew University of Jerusalem, Jerusalem 91120, Israel; Noam.Steinman@mail.huji.ac.il (N.Y.S.); noambent@gmail.com (N.Y.B.)

* Correspondence: avid@ekmd.huji.ac.il

Received: 1 October 2020; Accepted: 12 October 2020; Published: date

**^1^H NMR spectra of polymers I-IX**

**
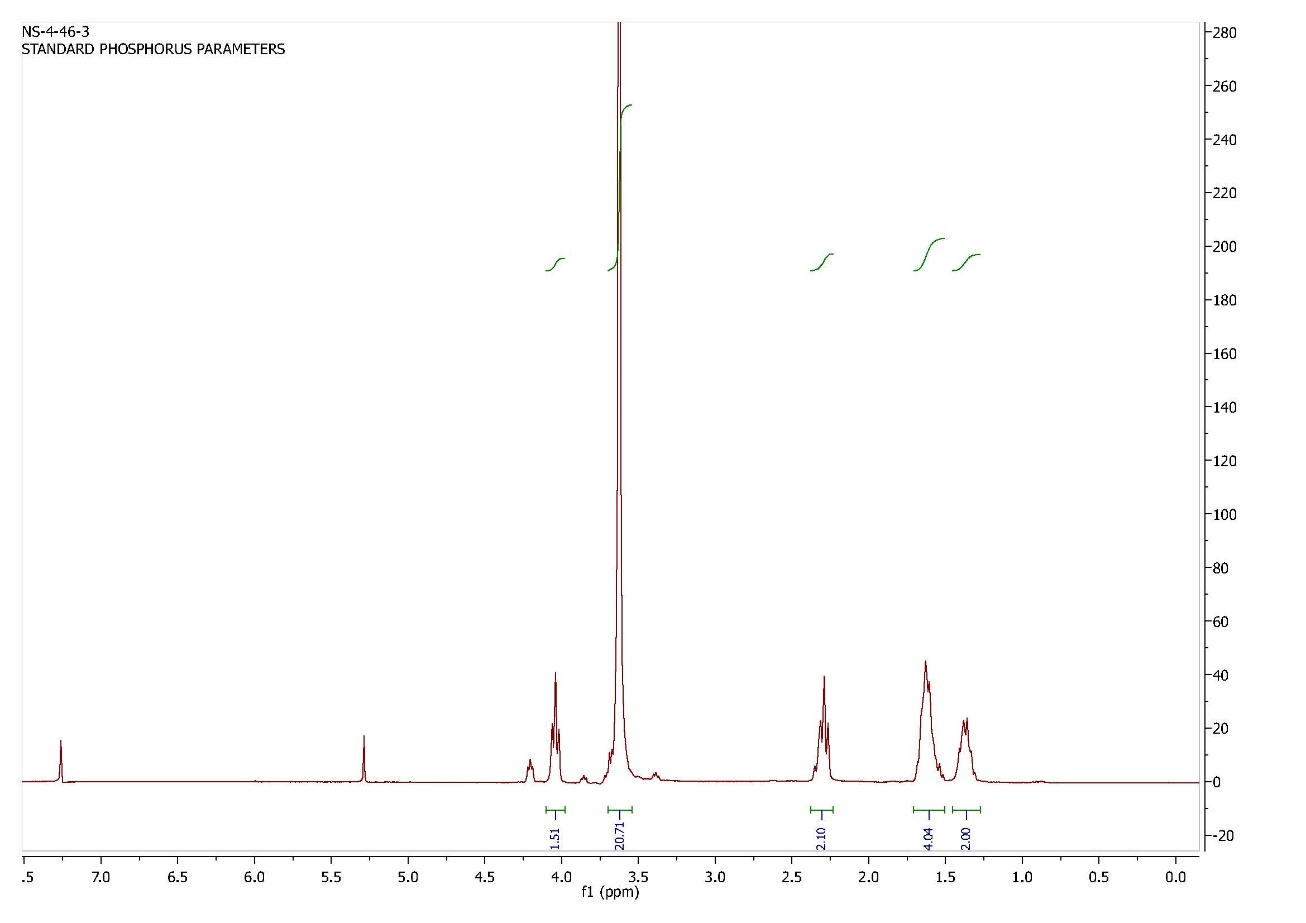
**

**Figure S1.** ^1^H NMR spectrum of **I**

**
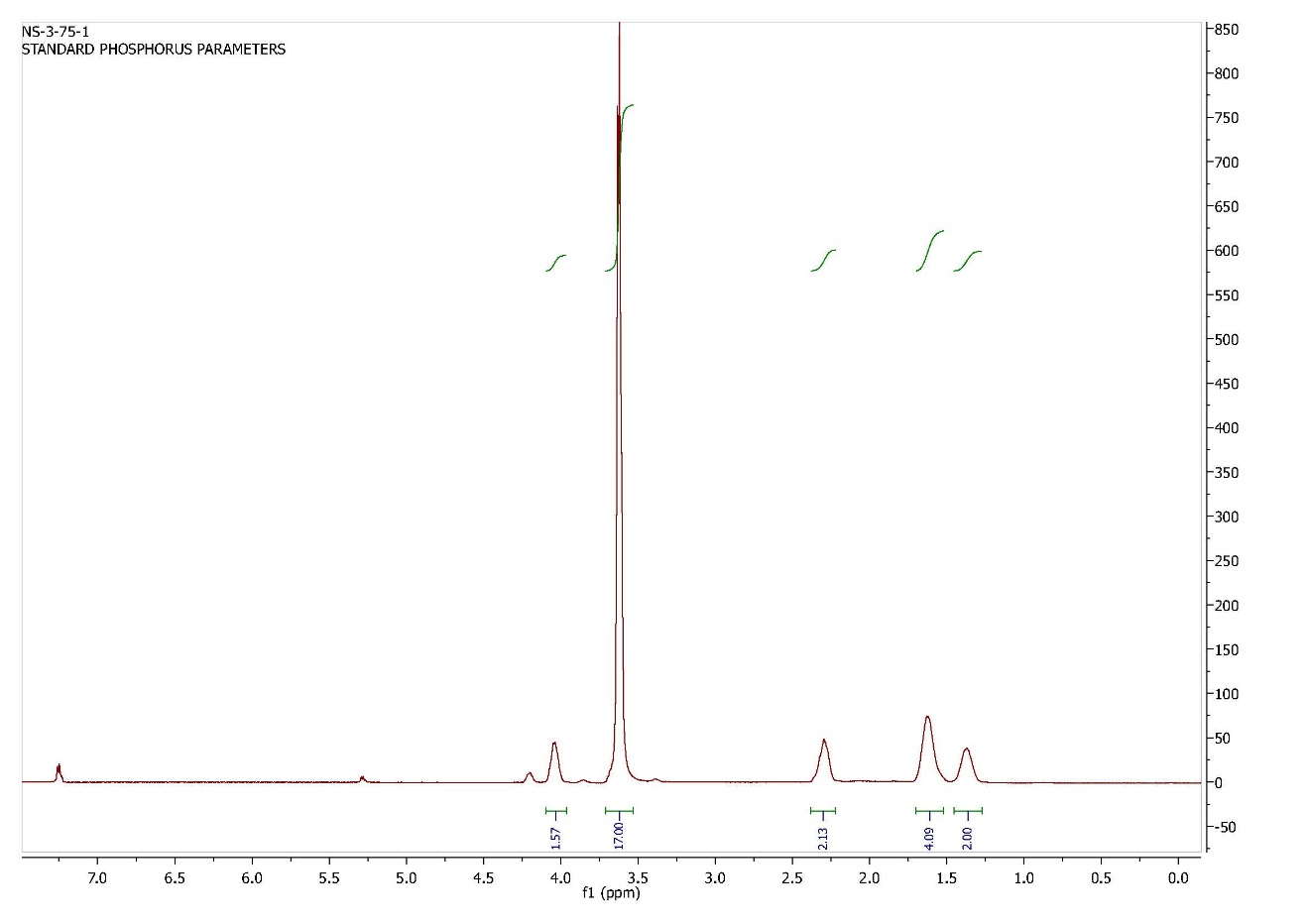
**

**Figure S2.** ^1^H NMR spectrum of **II**

**
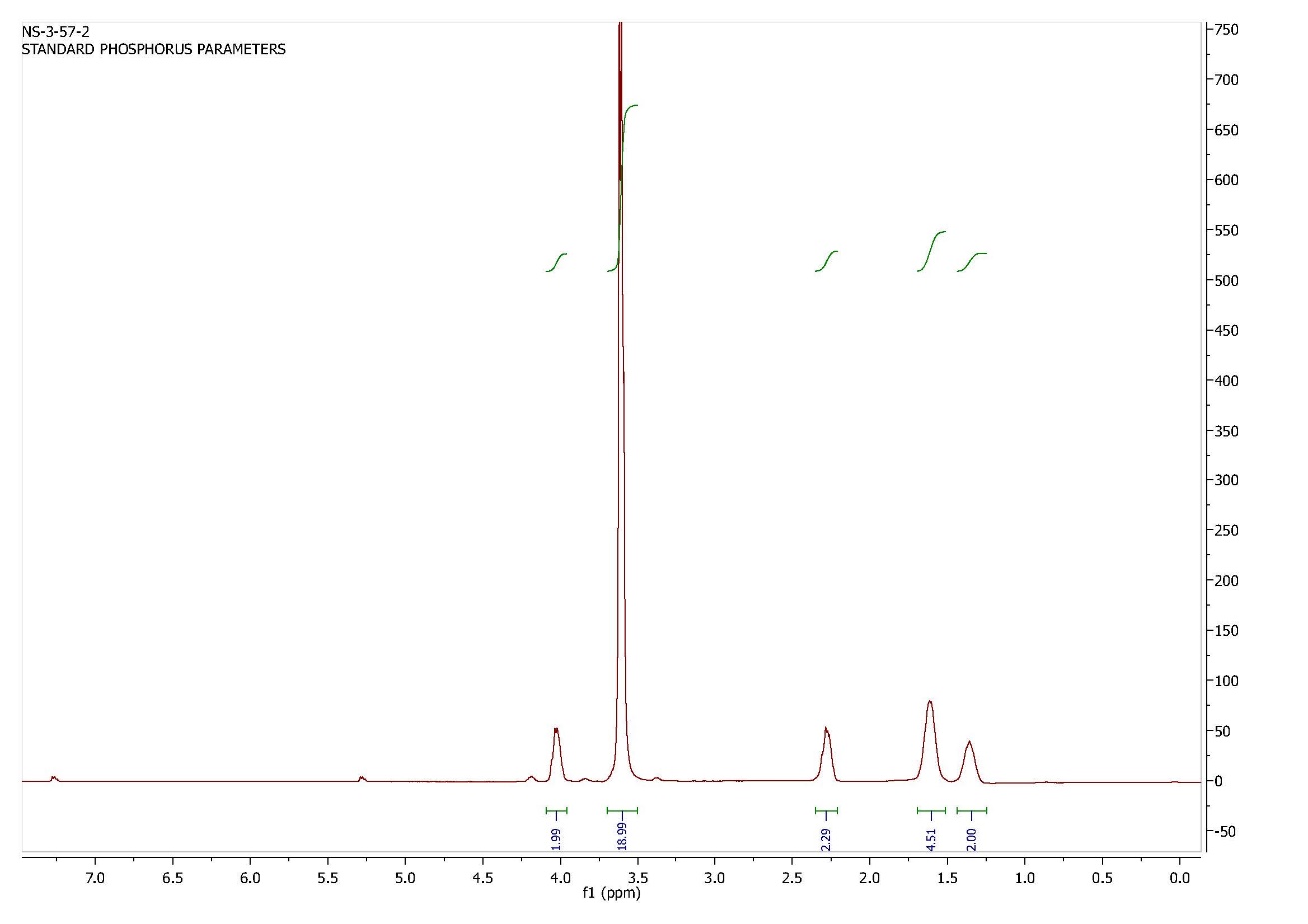
**

**Figure S3.** ^1^H NMR spectrum of **III**

**
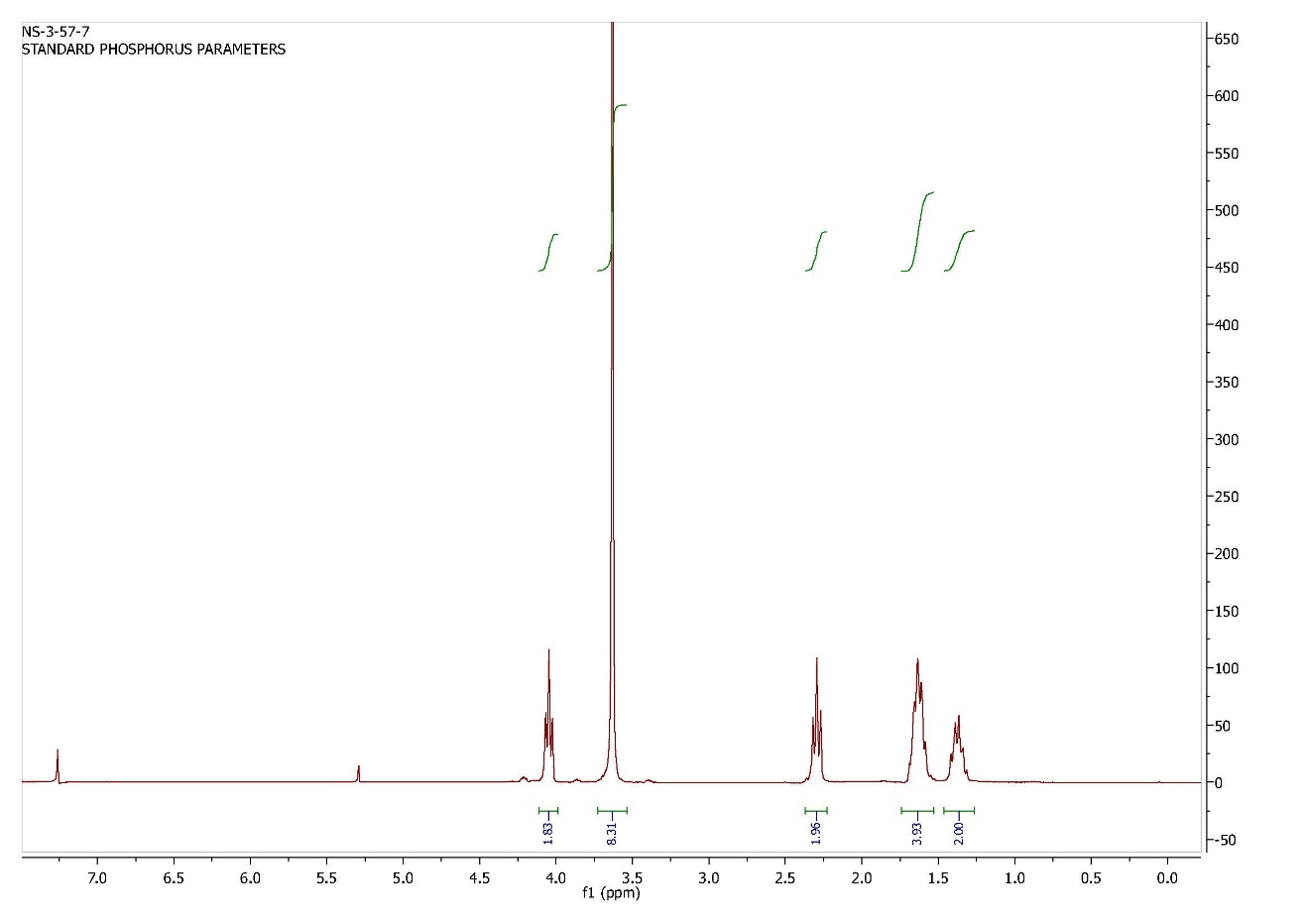
**

**Figure S4.** ^1^H NMR spectrum of **IV**

**
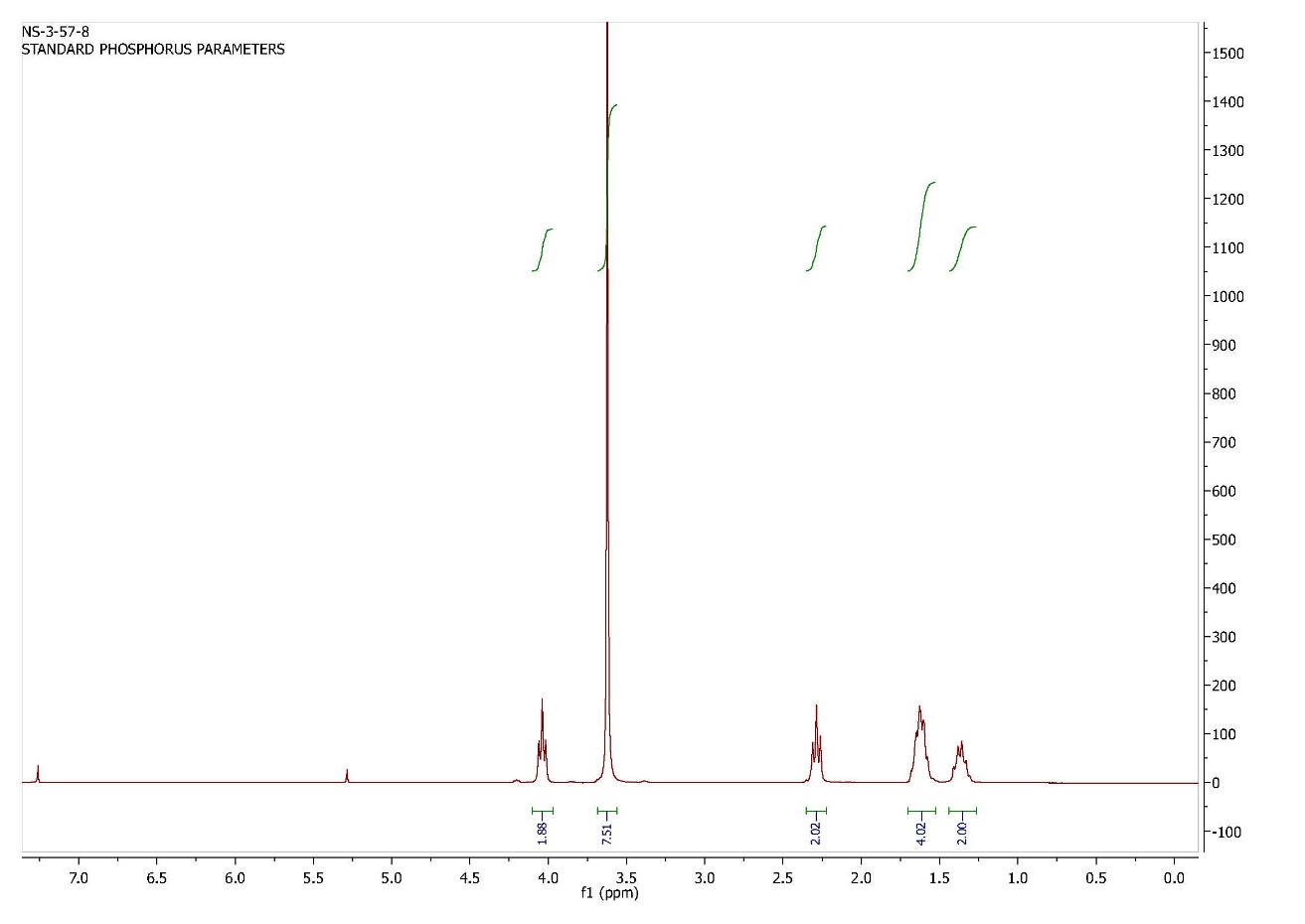
**

**Figure S5.** ^1^H NMR spectrum of **V**

**
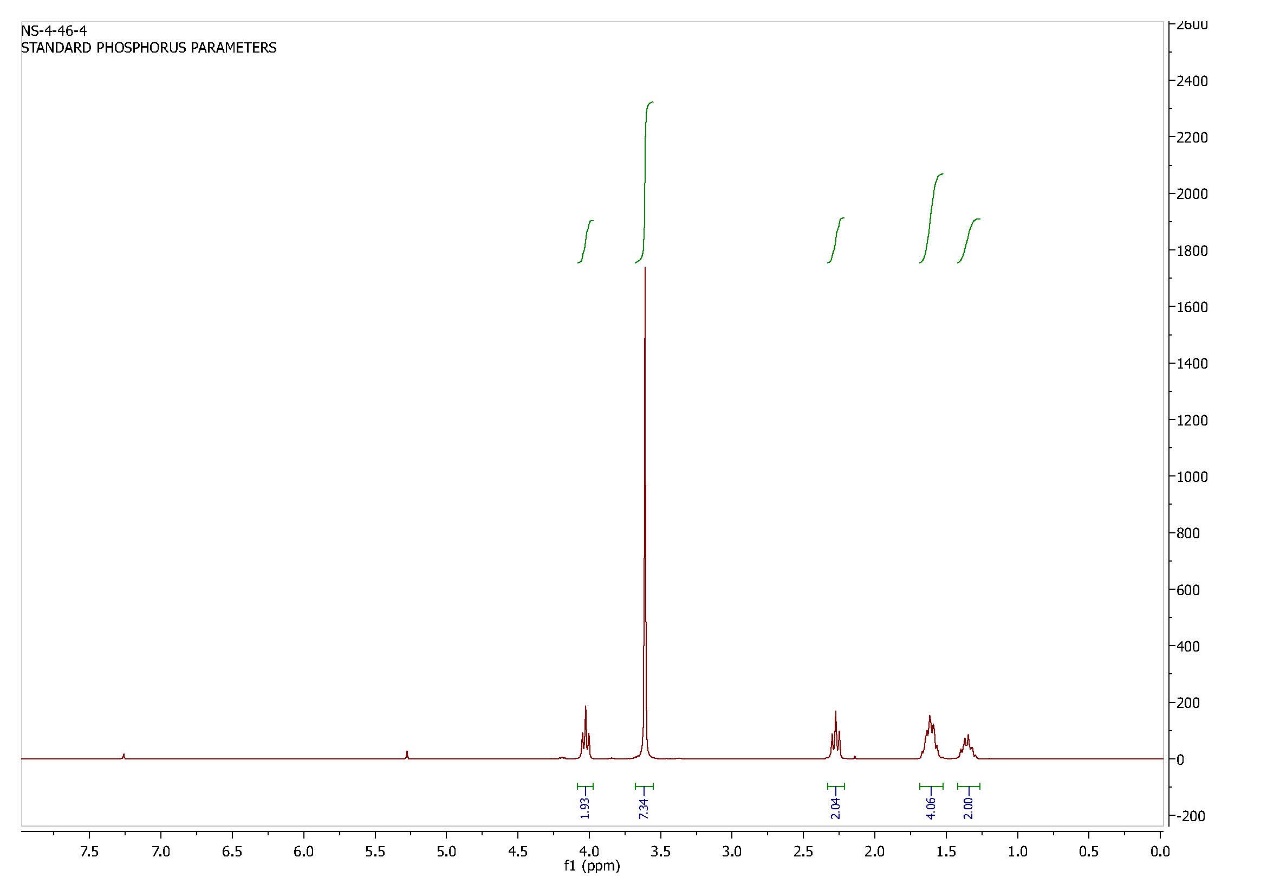
**

**Figure S6.** ^1^H NMR spectrum of **VI**

**
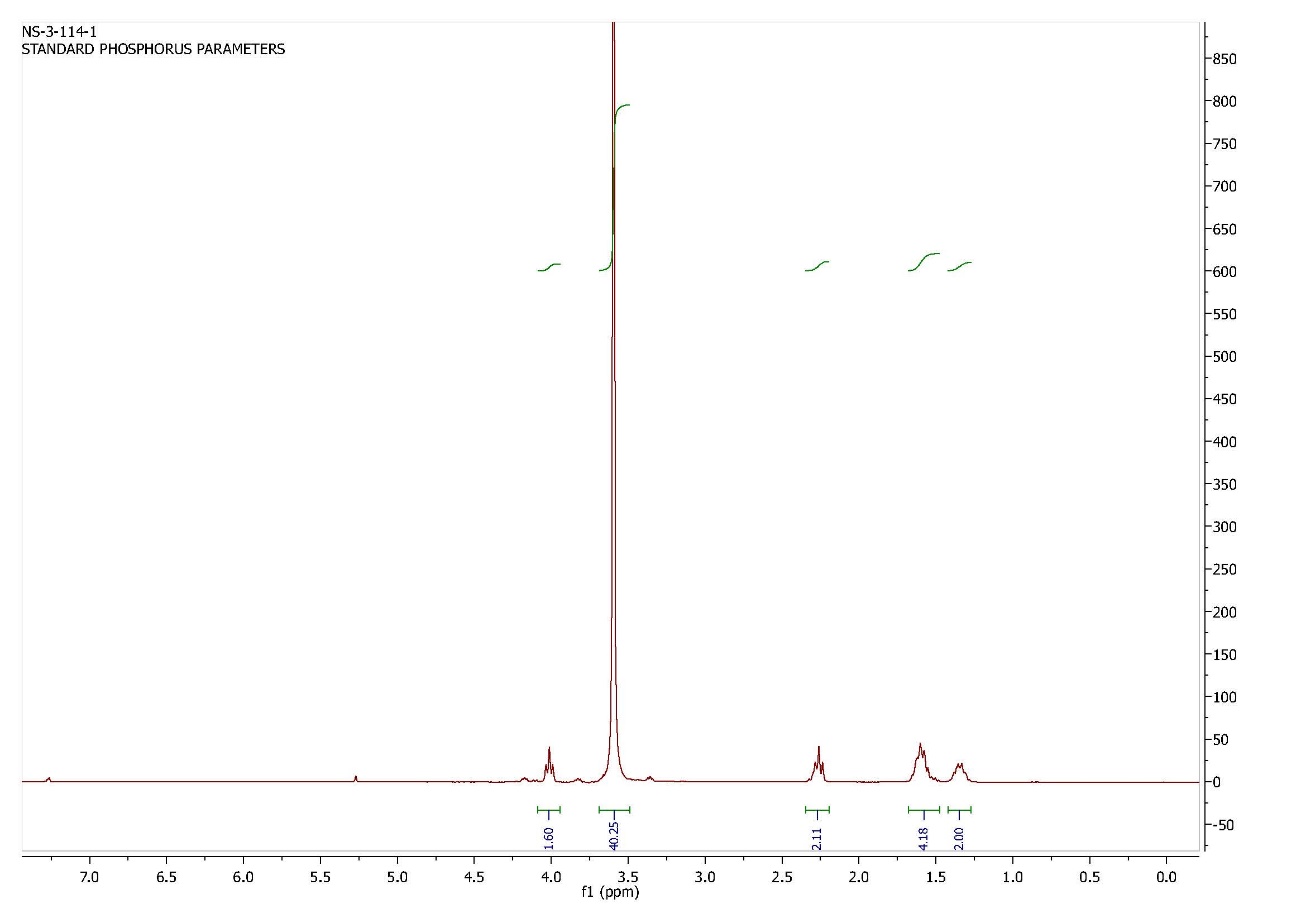
**

**Figure S7.** ^1^H NMR spectrum of **VII**

**
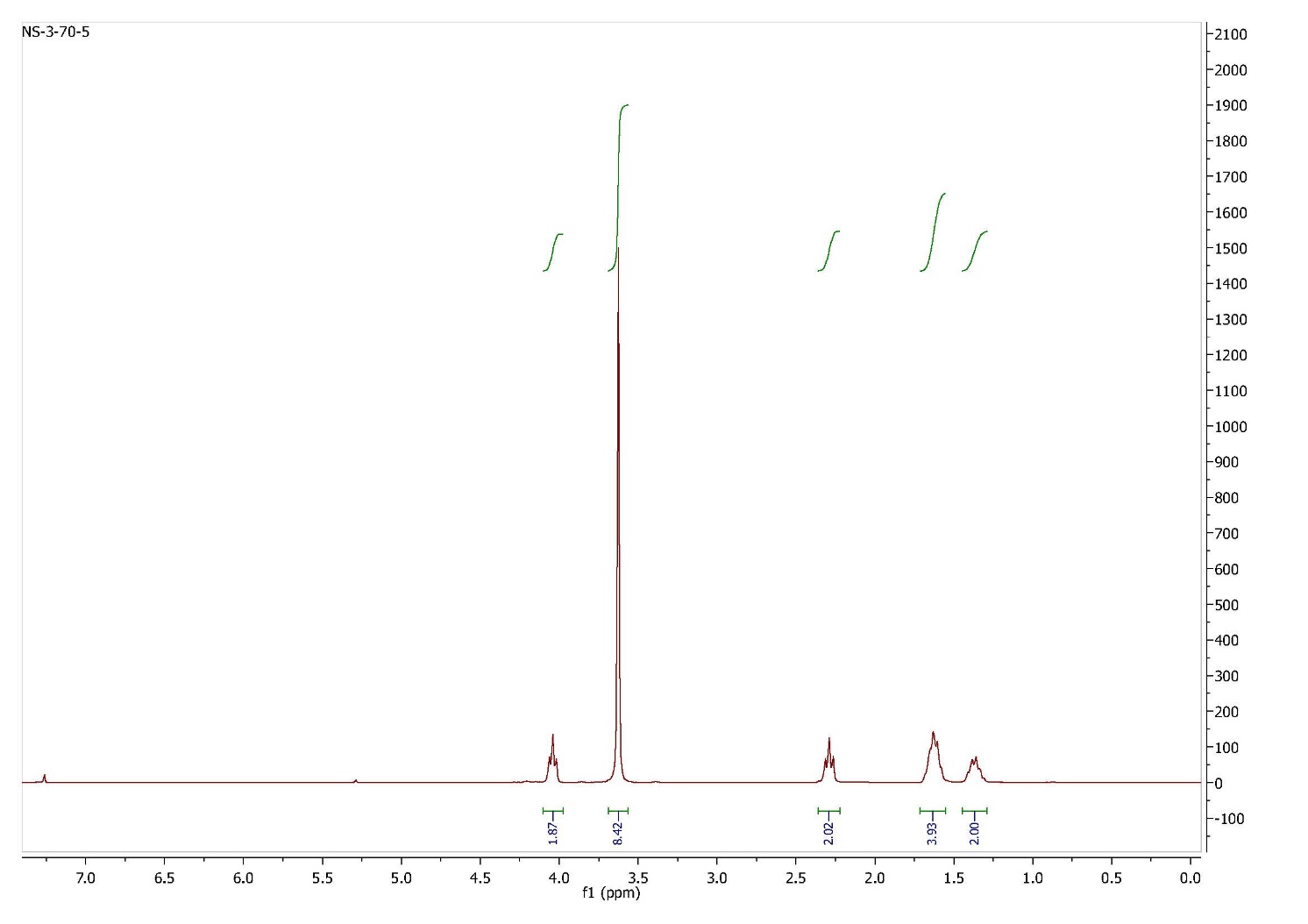
**

**Figure S8.** ^1^H NMR spectrum of **VIII**

**
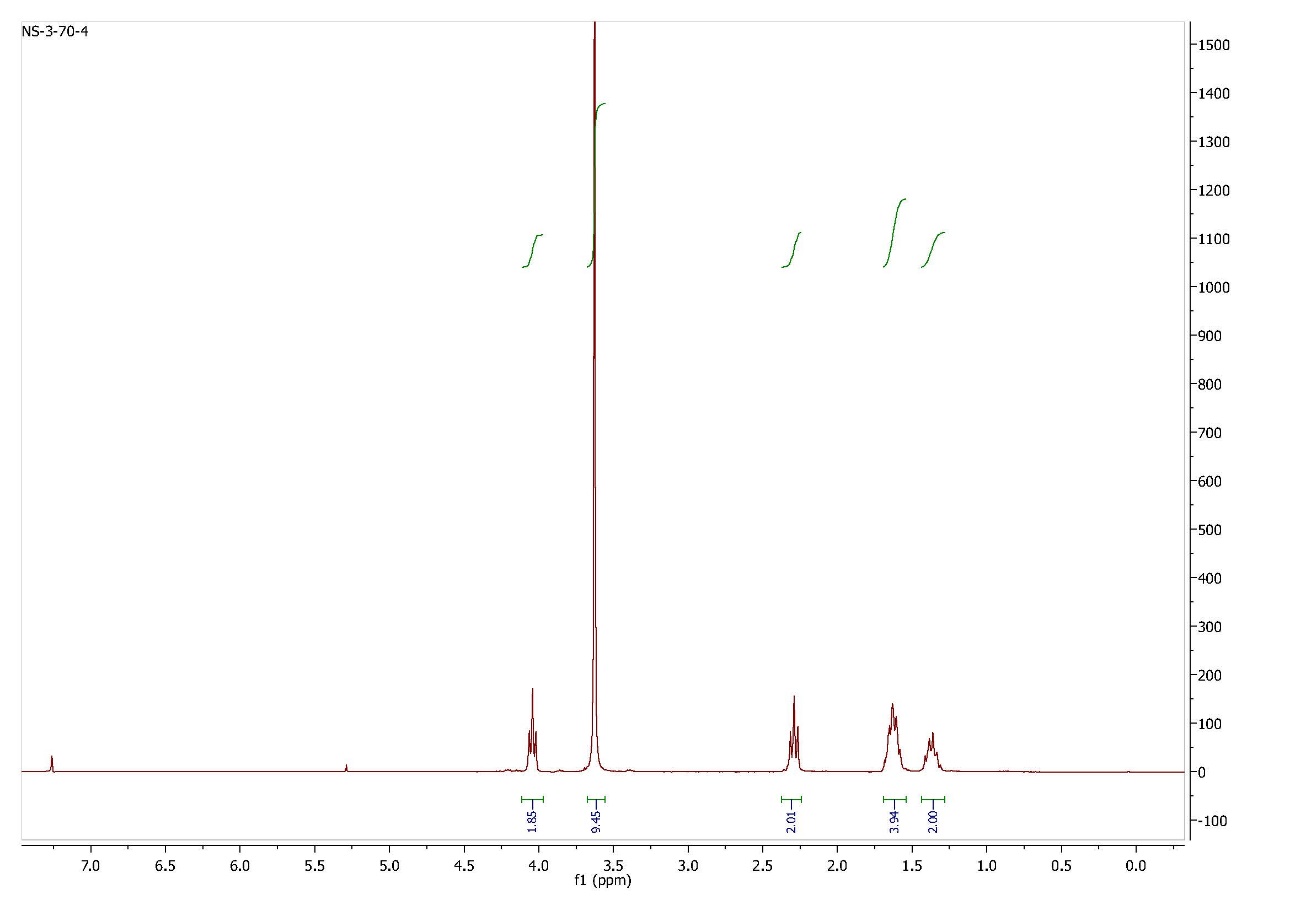
**

**Figure S9.** ^1^H NMR spectrum of **IX**

**IR spectra of polymers I-IX**

**
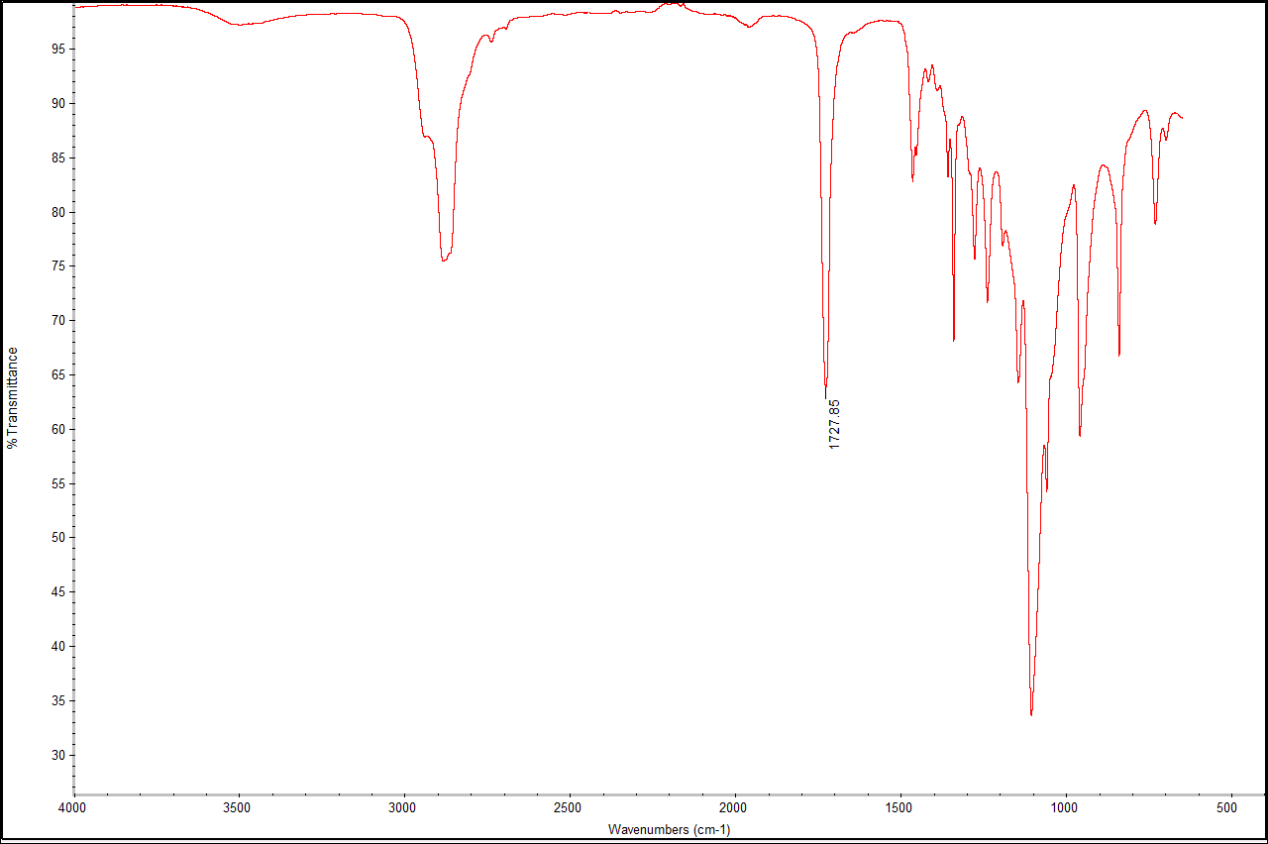
**

**Figure S10.** IR spectrum of **I** with labelled carbonyl stretch.

**
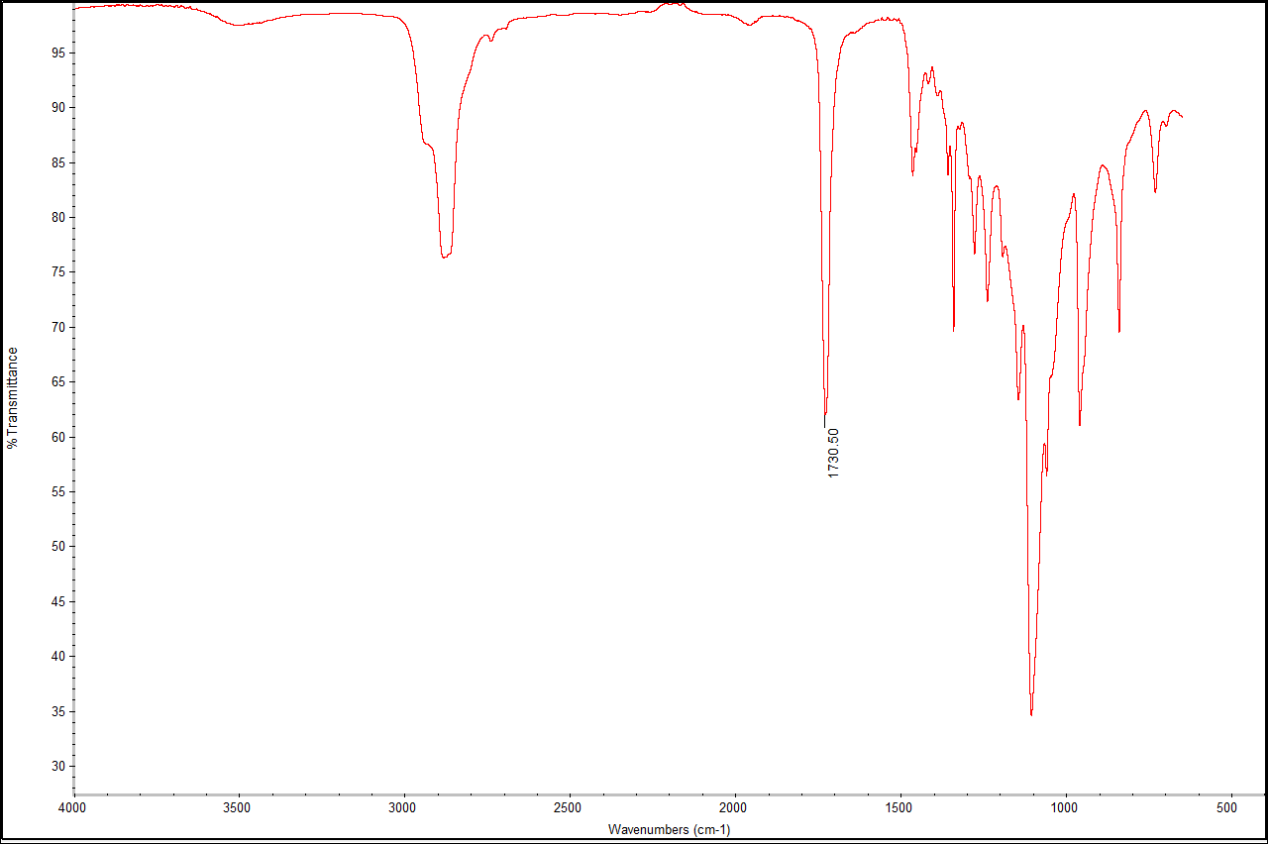
**

**Figure S11.** IR spectrum of **II** with labelled carbonyl stretch.

**
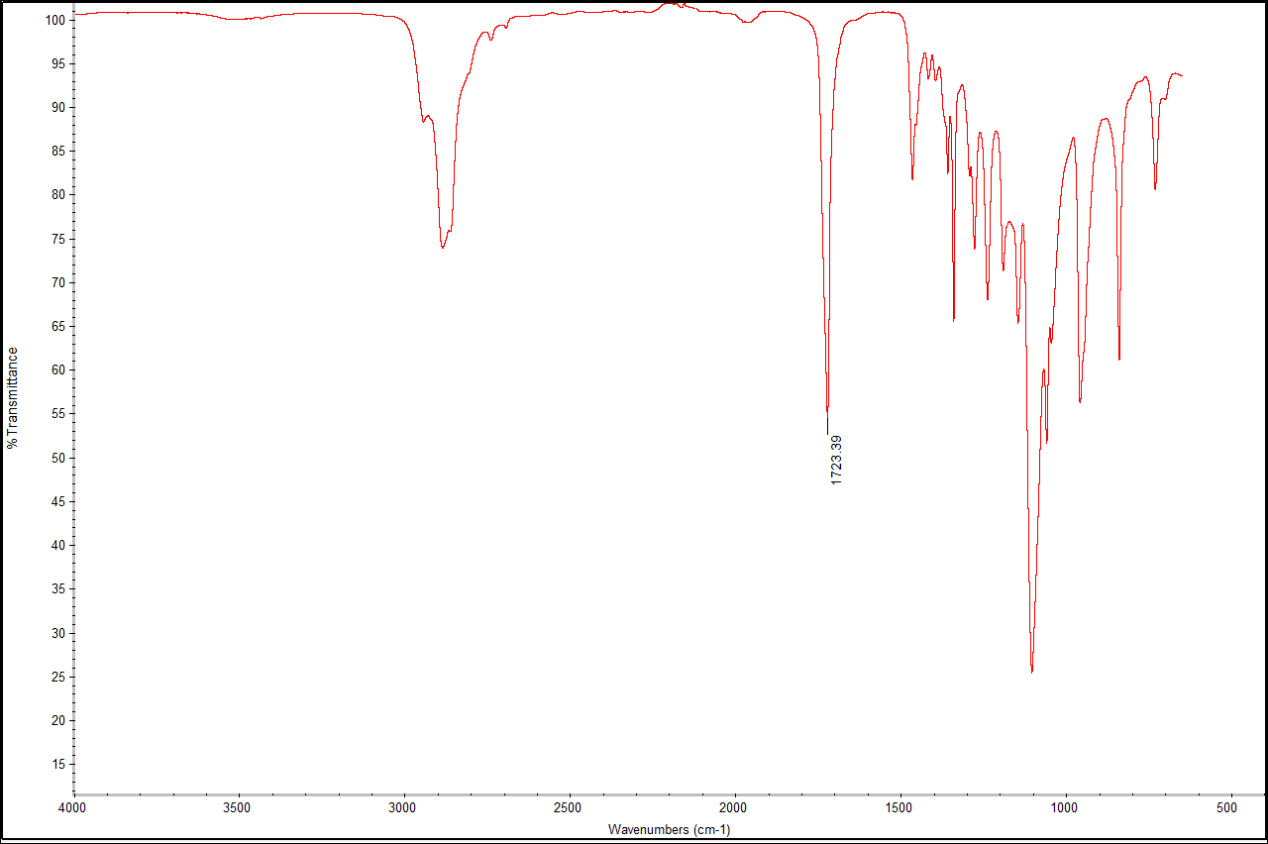
**

**Figure S12.** IR spectrum of **III** with labelled carbonyl stretch.

**
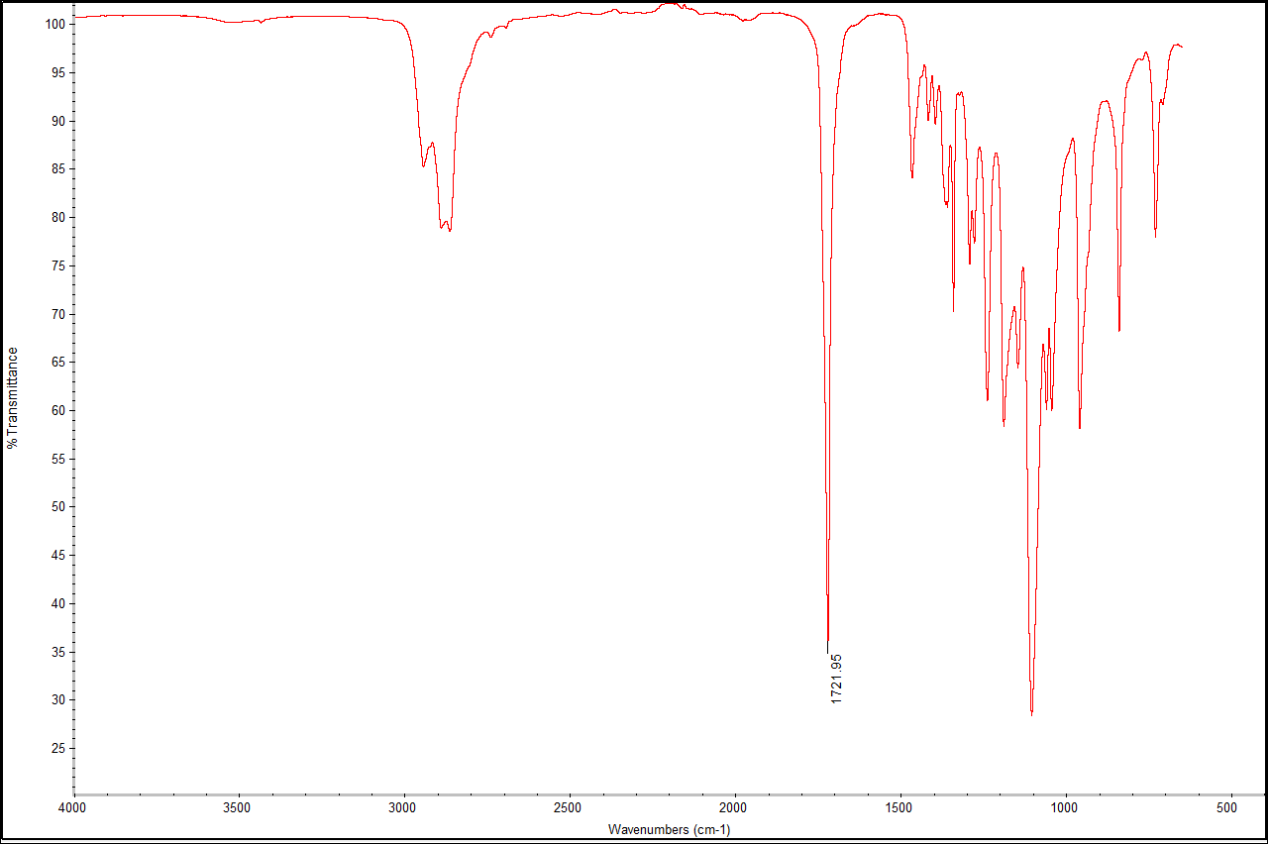
**

**Figure S13.** IR spectrum of **IV** with labelled carbonyl stretch.

**
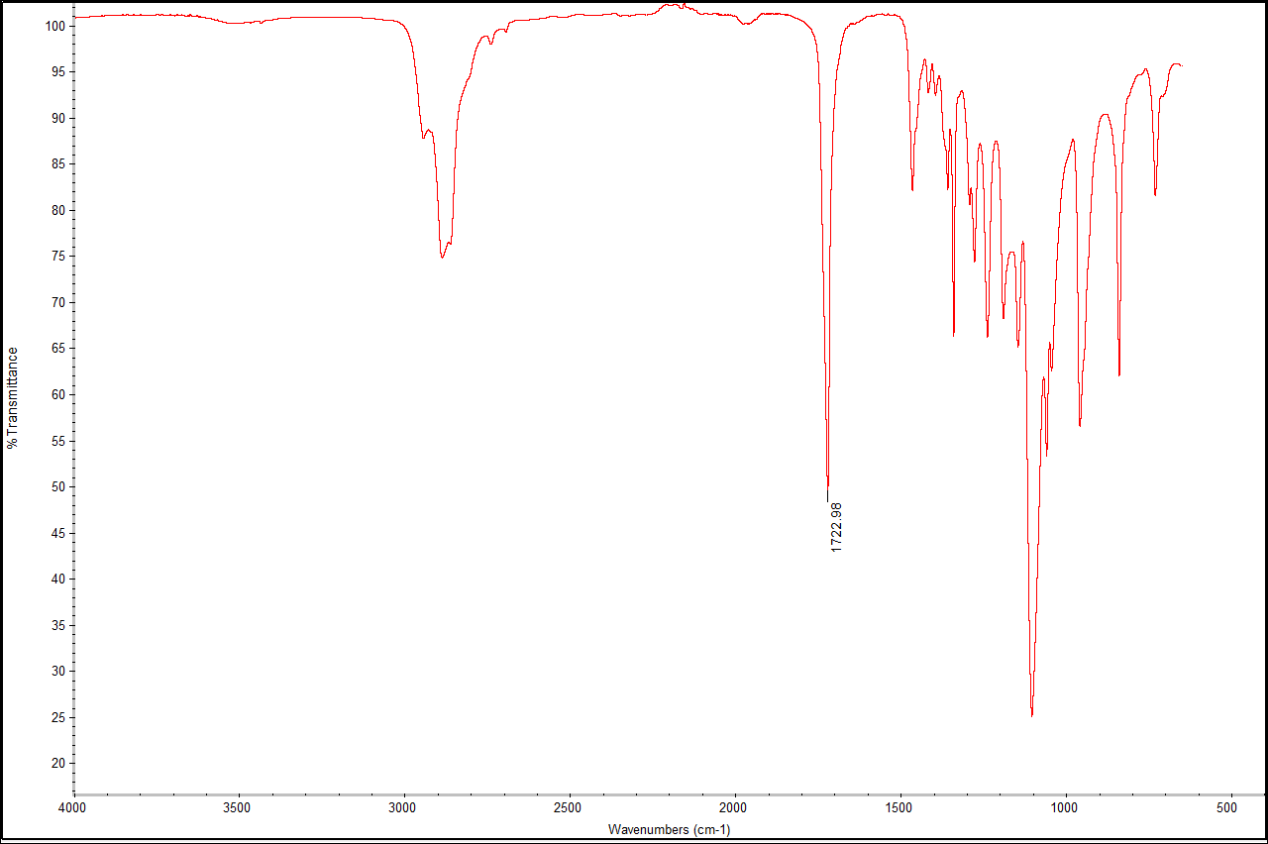
**

**Figure S14.** IR spectrum of **V** with labelled carbonyl stretch.

**
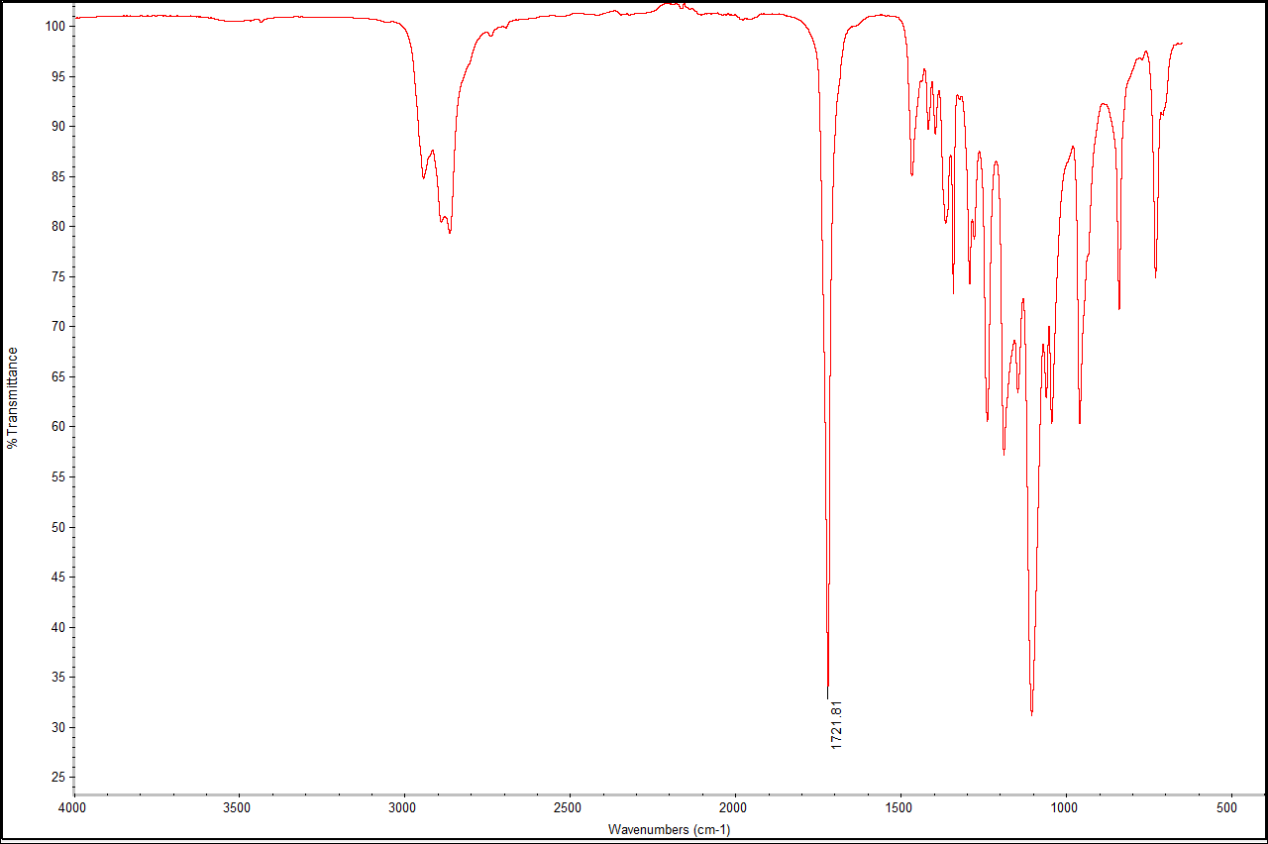
**

**Figure S15.** IR spectrum of **VI** with labelled carbonyl stretch.

**
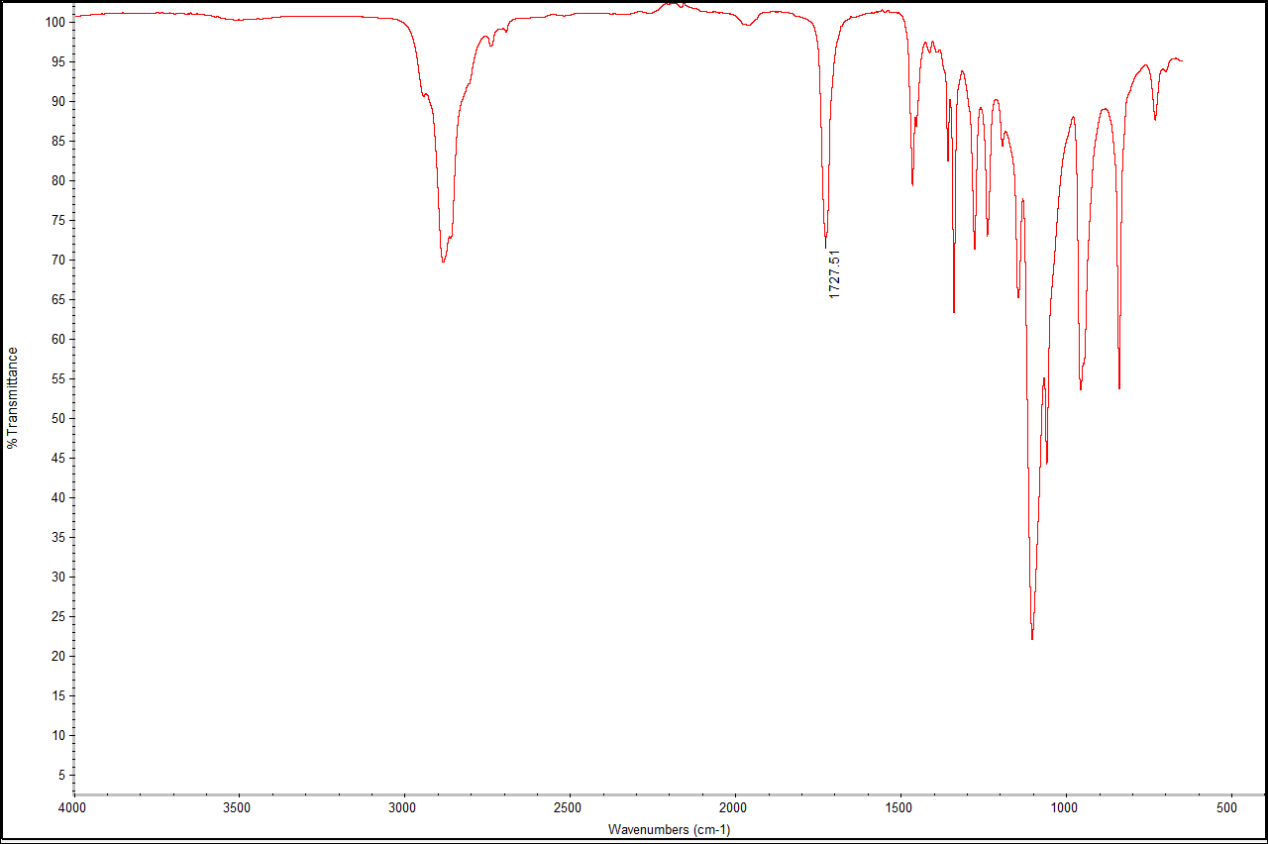
**

**Figure S16.** IR spectrum of **VII** with labelled carbonyl stretch.

**
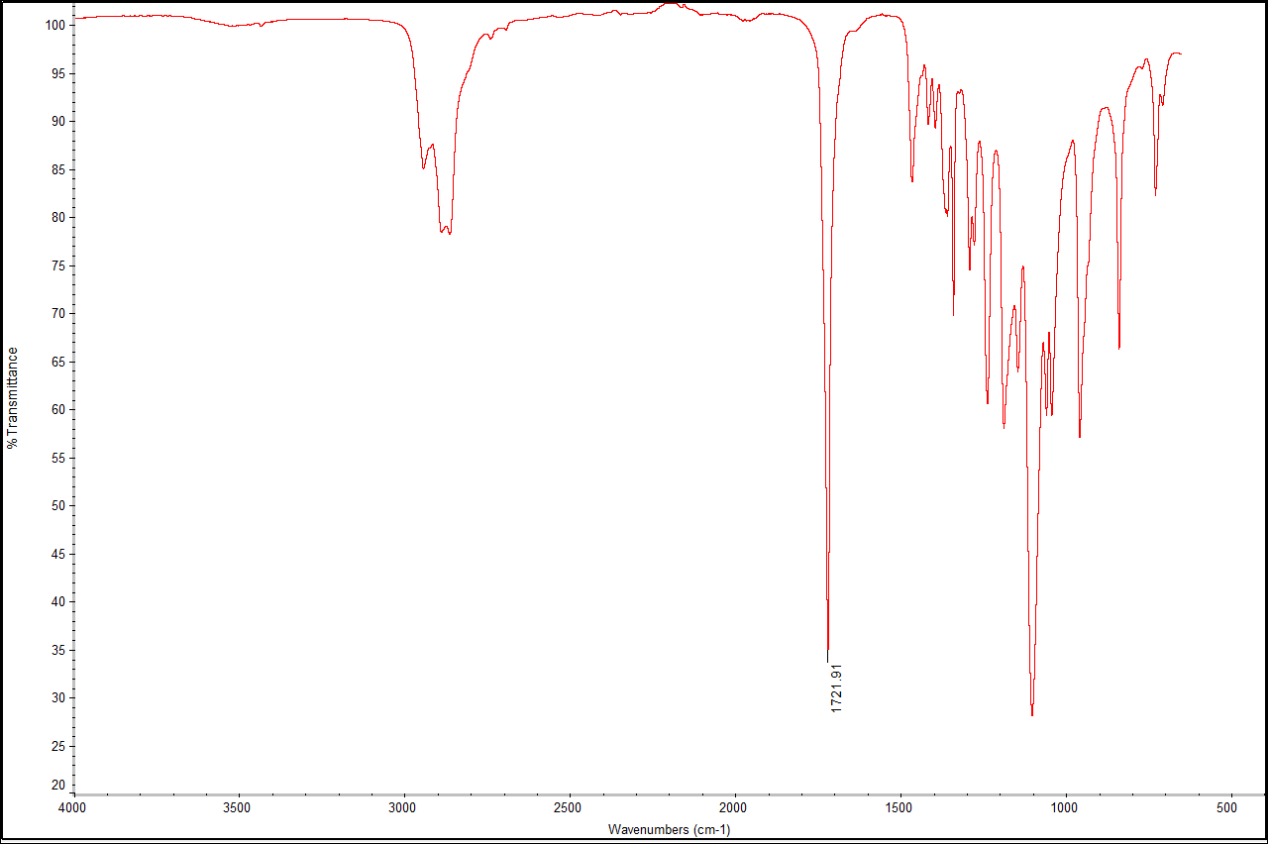
**

**Figure S17.** IR spectrum of **VIII** with labelled carbonyl stretch.

**
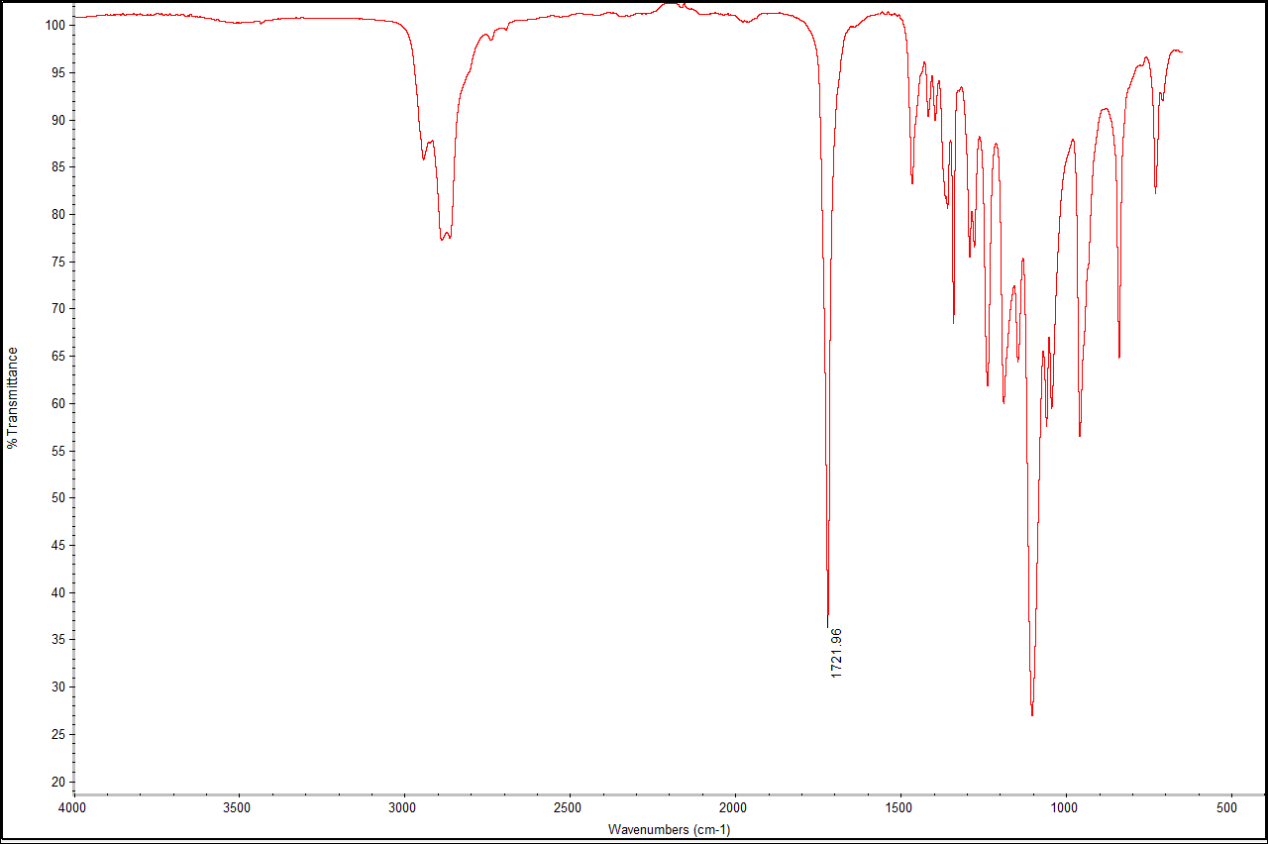
**

**Figure S18.** IR spectrum of **IX** with labelled carbonyl stretch.

**GPC chromatographs of polymers I-X**


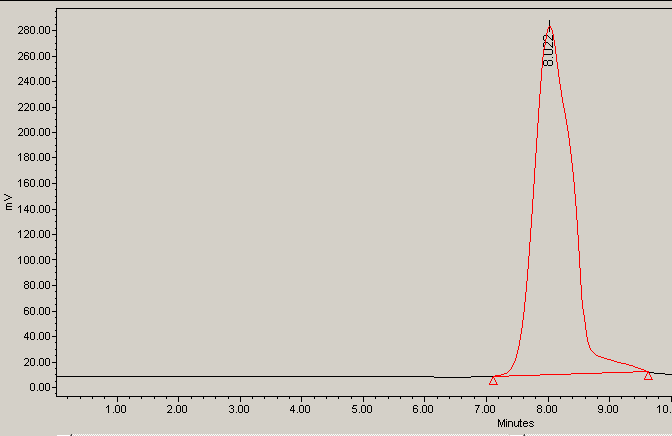


**Figure S19.** GPC chromatograph of **I** with retention time displayed.


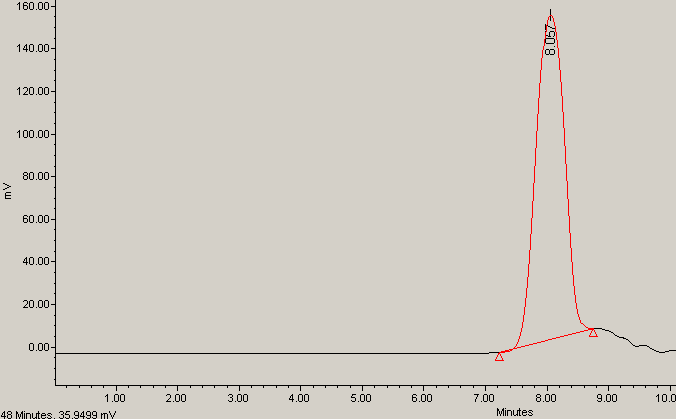


**Figure S20.** GPC chromatograph of **II** with retention time displayed.


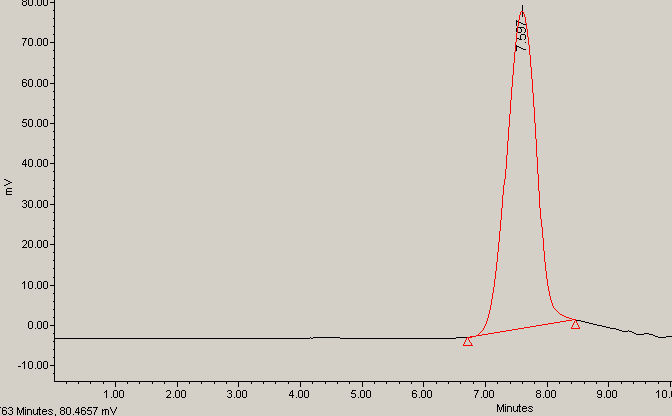


**Figure S21.** GPC chromatograph of **III** with retention time displayed.


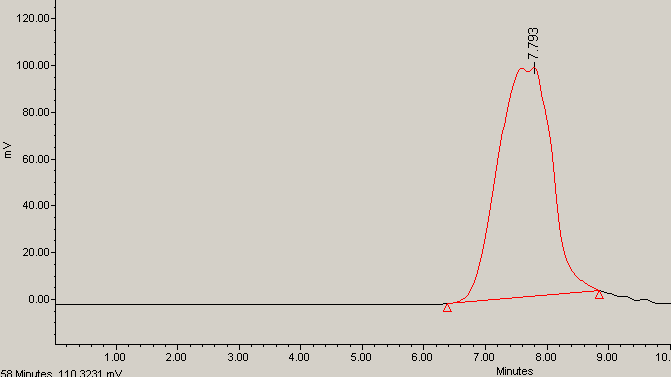


**Figure S22.** GPC chromatograph of **IV** with retention time displayed.**Figure S23.** GPC chromatograph of **V** with retention time displayed.


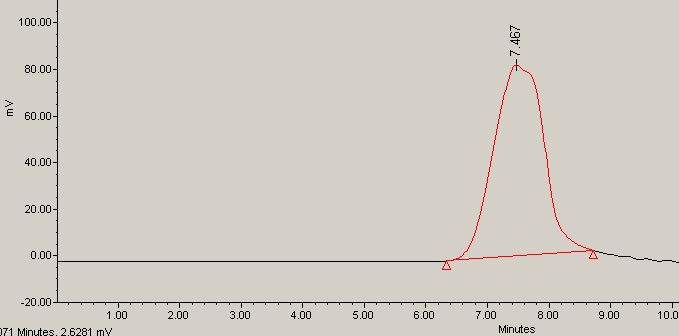

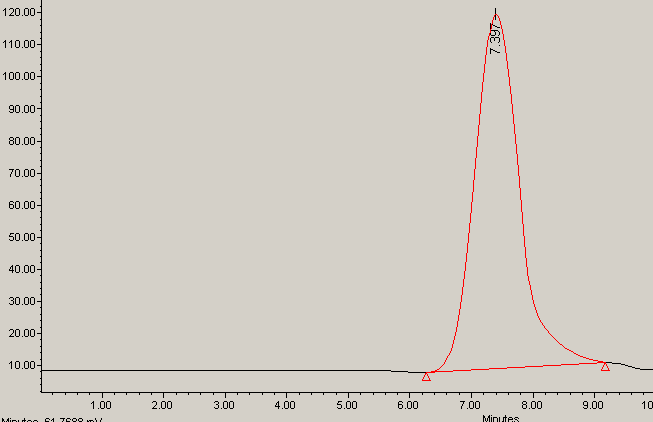


**Figure S24.** GPC chromatograph of **VI** with retention time displayed.


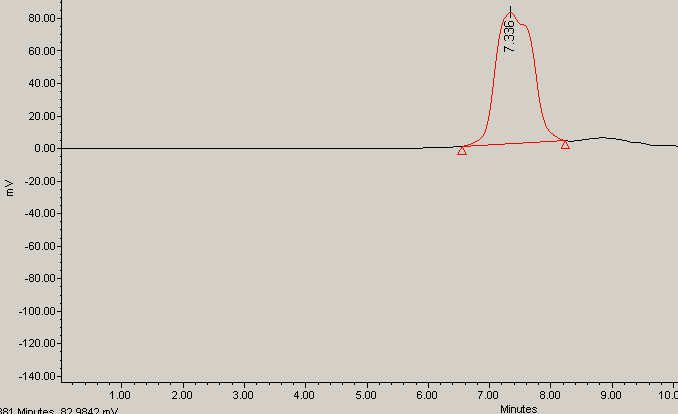


**Figure S25.** GPC chromatograph of **VII** with retention time displayed.


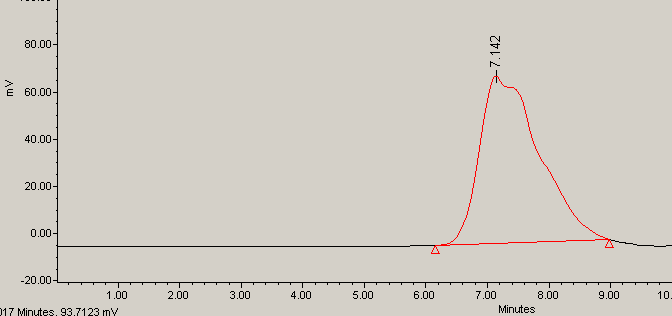


**Figure S26.** GPC chromatograph of **VIII** with retention time displayed.


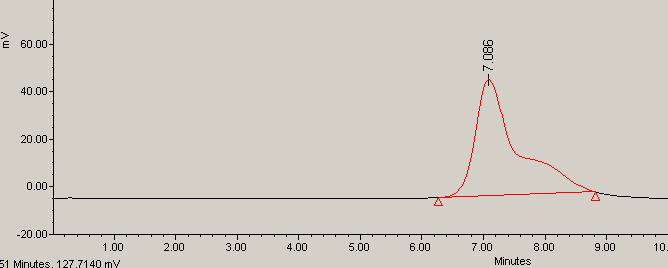


**Figure S27.** GPC chromatograph of **IX** with retention time displayed.


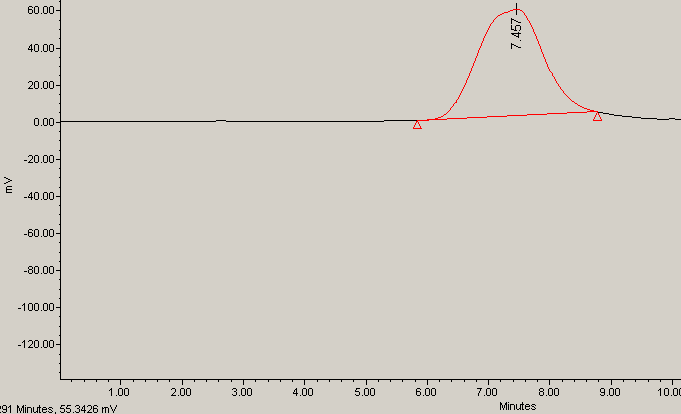


**Figure S28.** GPC chromatograph of **X** with retention time displayed.
